# Low-kV high-pitch one-scan aortic and coronary CT angiography using dual-source photon-counting CT: comparison with separate acquisition strategies

**DOI:** 10.1186/s12880-026-02629-0

**Published:** 2026-07-31

**Authors:** Hiroaki Kato, Suguru Araki, Satoshi Nakamura, Takafumi Ouchi, Akio Yamazaki, Takatoshi Higashigawa, Noriyuki Kato, Yasutaka Ichikawa, Hajime Sakuma, Kakuya Kitagawa

**Affiliations:** 1 Department of Radiology, Mie University Hospital, 2-174 Edobashi, Tsu, Mie 514-8507 Japan; 2 Department of Advanced Diagnostic Imaging, Mie University Graduate School of Medicine, 2-174 Edobashi, Tsu, Mie 514-8507 Japan; 3 MUDX Initiative, Mie University Organization for Research Initiative and Promotion, 1577 Kurimamachiyacho, Tsu, Mie 514-8507 Japan

**Keywords:** Coronary CT angiography, CCTA, CT aortography, Photon counting detector CT, PCD-CT, Cardiovascular disease

## Abstract

**Background:**

For patients with aortic diseases, coronary CT angiography (CCTA) is performed for preoperative evaluation of coronary artery disease, often in combination with CT angiography of thoracic and abdominal aorta (CTAO) within the same examination. A dual-source photon-counting detector CT (PCD-CT) offers advantages including improved dose efficiency and reduction of electronic noise, which might help maintain image quality even at low tube voltages. This study aimed to compare a low-kV one-scan CCTA/CTAO protocol with a low-kV dedicated CCTA followed by a higher-kV CTAO acquisition (separate-scan) regarding radiation dose and image quality.

**Methods:**

This retrospective, single-center study included 42 patients who underwent CCTA and CTAO using PCD-CT for preoperative CAD screening for aortic disease: 19 had separate-scan, 23 had one-scan CCTA and CTAO. Image quality for CCTA was assessed using a four-point scale (excellent, good, fair, non-diagnostic). Image quality for CTAO was assessed using a five-point scale (excellent, good, moderate, poor, non-diagnostic).

**Results:**

Radiation dose was significantly lower in the one-scan group compared to the separate-scan group (mean total dose-length product, 175.8 ± 71.1 vs. 769.4 ± 212.1 mGy·cm; *P* < 0.001). For CCTA, subjective image quality scores were comparable between the two groups (*P* = 0.537), although the separate-scan group showed significantly higher objective CNR compared to the one-scan group (*P* =0.025). For CTAO, the subjective image quality was significantly superior in the one-scan group (*P* < 0.01), despite comparable CNR between the groups (*P* = 0.13). The proportions of good or excellent ratings were 97.1% and 98.4% for CCTA, and 85.1% and 91.3% for CTAO in the separate-scan and one-scan groups, respectively.

**Conclusions:**

The low-kV one-scan strategy using PCD-CT substantially reduced radiation dose while maintaining comparable subjective CCTA quality and superior subjective CTAO quality. This approach may provide a dose-efficient option in selected patients requiring primary aortic assessment with concomitant coronary screening; however, its adequacy for preoperative CAD screening remains to be established.

**Supplementary Information:**

The online version contains supplementary material available at https://doi.org/10.1186/s12880-026-02629-0.

## Introduction

Aortic diseases are a leading cause of mortality worldwide [1] and frequently coexist with coronary artery disease (CAD) as manifestations of systemic atherosclerosis [2]. In patients with aortic disease scheduled for surgical or endovascular intervention, assessment of concomitant CAD is clinically important for perioperative risk stratification [3]. Coronary computed tomography angiography (CCTA) has become a widely used noninvasive alternative to invasive coronary angiography, and in clinical practice it is often performed together with CT angiography of the aorta (CTAO) as part of the preoperative workup.

In such combined examinations, the thoracoabdominal CTAO component often accounts for a substantial proportion of overall radiation exposure because of its long z-axis coverage. Therefore, meaningful dose reduction is likely to depend primarily on optimization of the aortic acquisition strategy. Recent studies have shown that ECG-gated high-pitch thoracoabdominal CTAO using dual-source photon-counting detector CT (PCD-CT) is feasible and can provide favorable image quality relative to conventional energy-integrating detector CT (EID-CT) [4, 5]. However, these studies have largely evaluated PCD-CT under standard or near-standard tube-voltage conditions and have mainly emphasized spectral capabilities, such as low-keV virtual monoenergetic imaging, to improve contrast conspicuity or salvage suboptimal enhancement. From the perspective of further radiation-dose reduction, an important next question is whether the acquisition itself can be shifted toward a lower tube-voltage strategy.

Lower tube-voltage imaging is attractive for dose reduction, but it is often constrained by increased image noise in EID-CT. PCD-CT may be particularly advantageous in this setting because its improved dose efficiency and reduced electronic noise can help preserve image quality at low tube voltage. This concept has already been demonstrated for CCTA; Araki et al. reported ultra-low-dose CCTA can be achieved using low-tube-voltage, high-pitch acquisition with dual-source PCD-CT while maintaining diagnostic image quality [6].

Building on these findings, an attractive approach is to combine CCTA and CTAO within a single ECG-synchronized, low-tube-voltage, high-pitch acquisition. This one-scan strategy is expected to reduce radiation dose and simplify clinical workflow by eliminating the need for separate CCTA and CTAO acquisitions. However, its clinical value depends on both aortic and coronary image quality. Two key issues remain. First, although ECG-gated high-pitch thoracoabdominal CTAO using PCD-CT has been reported, aortic image quality at low-tube-voltage has not been investigated. Second, concomitant coronary image quality in such an aorta-oriented acquisition has not been sufficiently investigated. If coronary image quality is insufficient, a dedicated CCTA would still be required for CAD screening, thereby negating the advantages of dose reduction and workflow simplification.

Accordingly, the present study compared two examination strategies on dual-source PCD-CT: (1) a low-kV, ECG-gated high-pitch one-scan protocol for combined coronary and thoracoabdominal aortic imaging, and (2) a conventional strategy consisting of dedicated CCTA followed by separate standard-helical CTAO at higher tube voltage. The primary objective was to compare radiation dose between the two strategies. The secondary objectives were to compare aortic image quality and to determine whether coronary image quality in the one-scan protocol was sufficient compared with dedicated CCTA.

## Materials and methods

### Study design

**Fig. 1:**
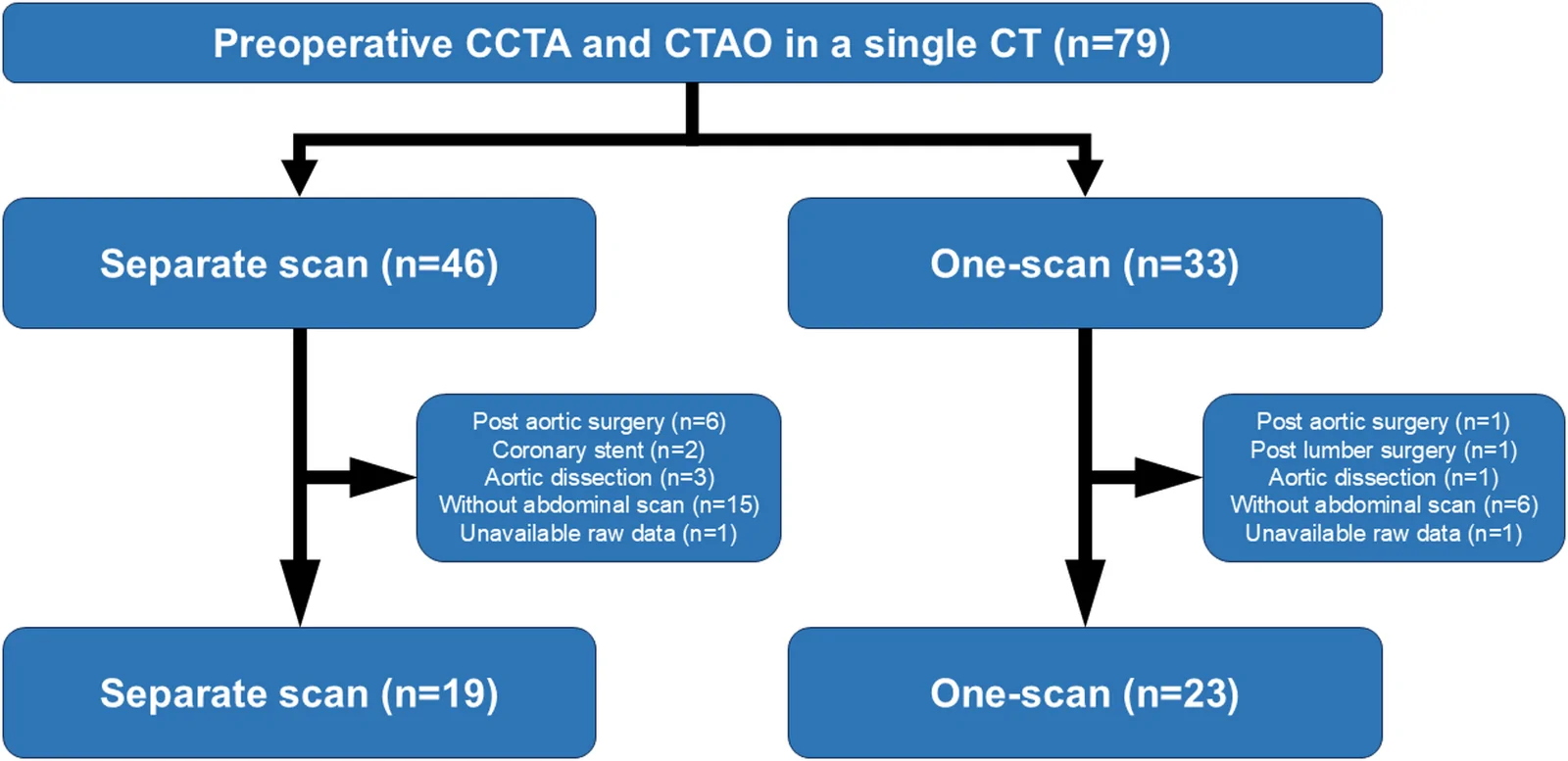
Study flowchart. Between August 2023 and July 2025, 79 consecutive patients who underwent both CCTA and CTAO for preoperative evaluation of cardiovascular disease in a single CT examination were initially considered for inclusion. Patients with prior coronary stent placement, aortic surgery and double barrel-aortic dissection, or significant metal artifacts (e.g., lumbar surgery) were excluded. Patients without plain CT or whose CTAO did not include the region from the abdomen to the pelvis were also excluded. Patients whose raw data were unavailable for specific keV reconstruction were also excluded. As a result, 19 underwent CCTA and CTAO in separate scans, while 23 underwent both CCTA and CTAO in a single scan. CCTA, coronary computed tomography angiography; CTAO, computed tomography angiography of the aorta

Between August 2023 and July 2025, 79 consecutive patients who underwent both CCTA and CTAO for preoperative evaluation of cardiovascular disease in a single CT examination were initially considered for this retrospective, single-center study. Of these, 46 patients underwent CCTA and CTAO as separate acquisitions, whereas 33 patients underwent a one-scan protocol. The choice of protocol was based on the referring physician’s request: the separate-scan protocol was selected when the primary clinical indication was CAD evaluation, whereas the one-scan protocol was used when comprehensive aortic assessment, including evaluation of the access route, was the main objective. Patients with a prior coronary stent placement, prior aortic surgery, double-barrel aortic dissection, or significant metal artifacts (e.g., lumbar surgery) were excluded. Patients without plain CT or whose CTAO did not include the region from the abdomen to the pelvis were excluded. Patients whose raw data were unavailable for specific keV reconstruction were also excluded. As a result, 19 patients were included in the separate-scan group and 23 in the one-scan group (Fig. 1).

This retrospective study was conducted in accordance with the Declaration of Helsinki. The study protocol was approved by the Clinical Research Ethics Review Committee of Mie University Hospital (approval no. H2019-207). The requirement for informed consent from patients was waived due to the retrospective nature of the study.

### Image acquisition

A first-generation dual-source PCD-CT system (NAEOTOM Alpha, Siemens Healthineers, Forchheim, Germany) was utilized for all CT examinations in the present study. During the procedure, patients were positioned in the supine position and monitored via electrocardiography (ECG) and automated blood pressure measurement. Initial anteroposterior and lateral scout images were obtained, followed by non-contrast CT for coronary artery calcium scoring. This pre-contrast scan was conducted using an ECG-triggered prospective high-pitch helical technique at 100 kVp with a tin (Sn) filter.

Regarding pharmacological preparation for both the one-scan and separate-scan protocols, 0.6 mg of sublingual nitroglycerin (Myocor spray; Toa Eiyo Co., Ltd, Tokyo, Japan) was given to each patient 5 min prior to the CCTA acquisition. Additionally, in cases where the heart rate remained above 60 beats per minute before CCTA, intravenous landiolol hydrochloride (Corebeta; Ono Pharmaceutical Co., Ltd, Osaka, Japan) was administered for heart rate control.

#### One-scan protocol (simultaneous CCTA and CTAO)

CCTA and CTAO were acquired in a single ECG-triggered prospective high-pitch helical mode (Turbo Flash Chest Pain), with scan initiation timed so that the CCTA acquisition corresponded to 70% of the R–R interval. For CTAO, the scan range extended from just above the aortic arch to below the femoral artery bifurcation, whereas for CCTA, based on the preceding coronary artery calcium scan, the scan range was set from 1 cm above the left main coronary artery to 1 cm below the cardiac apex.

Contrast timing was determined using bolus tracking. Iopamidol (Iopamilon-370, Bayer AG, Leverkusen, Germany) was injected at a flow rate of 26 mg iodine/kg/s for 12 s, followed by a 20 mL saline flush. A circular region of interest (ROI) placed at the level of the iliac crest is dynamically tracked over time, and upon reaching blood pool enhancement of 150 HU, triggering occurs with CCTA acquisition after a fixed (5 s) delay [7]. The acquisition parameters of the one-scan protocol were as follows: a pitch factor of 3.2, collimation of 144 mm × 0.4 mm, gantry rotation time of 0.25 s, and tube voltage of 70 kVp (*n* = 7) or 90 kVp (*n* = 16). Automatic exposure control (CARE keV, Siemens Healthineers, Forchheim, Germany) was utilized. Tube current was set by the CT scanner automatically to achieve an Image quality (IQ) level of 54.

#### Separate-scan protocol (separate CCTA and CTAO acquisitions)

In the separate-scan protocol, CCTA was acquired using either ECG-triggered sequential scanning at 65–85% of the R–R interval (*n* = 8) or high-pitch helical scanning with a starting phase of 70% of the R–R interval (*n* = 11), with the scan technique selected based on the patient’s heart rate and cardiac rhythm at the time of acquisition. ECG-triggered sequential scanning was performed in patients whose heart rate was reduced to < 65 bpm after intravenous landiolol hydrochloride administration, whereas high-pitch helical scanning was used in patients whose heart rate remained > 65 bpm despite intravenous landiolol hydrochloride administration. The scan ranges of CCTA and CTAO were determined using the same criteria as those applied in the one-scan protocol (Supplementary Table 1).

CCTA was performed using iopamidol (Iopamilon-370) as the contrast medium, and contrast timing was determined by a test bolus technique. A test bolus was administered for 2 s at a flow rate corresponding to an iodine delivery rate of 26 mg iodine/kg/s, followed by a 3-second saline chaser at the same rate. A circular ROI was placed at the level of the ascending aorta and dynamically tracked over time to generate a cardiac output–dependent time–attenuation curve, which was used to estimate both the bolus arrival time and the peak enhancement time [8]. Subsequently, 26 mg iodine/kg/s of contrast medium for 12 s was injected, followed by a 20 mL saline chaser. CCTA acquisition was initiated after a predefined delay based on the time to peak derived from the test bolus time–attenuation curve, with an additional delay of 3 s for ECG-triggered sequential scanning and 5 s for ECG-triggered high-pitch helical scanning. The CCTA acquisition parameters were as follows: collimation of 144 × 0.4 mm, a gantry rotation time of 0.25 s, and a tube voltage of 70 or 90 kVp selected using CARE keV; the IQ level was set to 67.

CTAO was automatically initiated immediately after CCTA acquisition without operator intervention, with an effective delay of approximately 10 s depending on system preparation time [9]. The acquisition parameters of CTAO were as follows: a pitch factor of 1.5, collimation of 144 mm × 0.4 mm, gantry rotation time of 0.25 s, and tube voltage of 120 kVp or 140 kVp. Automatic exposure control (CARE keV, Siemens Healthineers, Forchheim, Germany) was utilized. Tube current was set by the CT scanner automatically to achieve an IQ level of 54.

### Image reconstruction

All images were reconstructed on a dedicated workstation (syngo.via, VB10; Siemens Healthineers, Forchheim, Germany). CCTA reconstructions were identical across protocols: VMI at 53 keV reconstructed at 70% of the R–R interval, slice thickness/increment 0.4/0.2 mm, matrix 512 × 512, quantum iterative reconstruction (QIR) level 4, kernel Bv64, and a reconstruction field of view restricted to the heart.

CTAO reconstructions were also identical across protocols: VMI at 70 keV with a slice thickness/increment of 1.0/1.0 mm, QIR level 3, kernel Qr40, matrix 512 × 512, and a reconstruction field of view adapted to body habitus.

### Subjective image quality analysis of CCTA

The visual quality of CCTA images was analyzed through a consensus review by two radiologists specializing in cardiovascular imaging, with 11 (S.N.) and 5 (S.A.) years of experience, respectively. They performed a comprehensive evaluation based on a 4-point Likert scale, taking into account essential diagnostic factors such as vessel sharpness, contrast, motion-related artifacts, and beam hardening. The scoring criteria were defined as follows: (1) non-diagnostic, where image quality precludes any morphological or plaque assessment; (2) fair, where morphologic assessment remained possible despite image-quality limitations; (3) good, where only slight image defects were present and assessment was not substantially impaired; and (4) excellent, where the coronary and plaque morphology could be assessed without difficulty (Supplementary Figure 1) [6]. Each coronary segment was individually graded according to the 18-segment model established by the Society of Cardiovascular Computed Tomography [10].

### Subjective image quality analysis of CTAO

Two radiologists, with 6 (H.K.) and 8 (T.O.) years of experience in aortic intervention and imaging, conducted a consensus reading to assess the subjective image quality of the CTAO data. They were blinded to the clinical indication, experimental allocation, and imaging results. For image quality assessment, the readers evaluated six predefined aortic segments: the ascending aorta, aortic arch, descending aorta, abdominal aorta, terminal aorta, and common iliac artery. Each segment was rated on a five-point Likert scale with the following criteria (1 = non-diagnostic, 2 = poor, 3 = moderate, 4 = good, 5 = excellent) (Supplementary Figure 2) [11]. Readers could adjust the window width and level settings manually.

### Objective image quality analysis of CCTA

To evaluate objective image quality, we quantified several metrics: signal intensity, image noise, contrast-to-noise ratio (CNR), and signal-to-noise ratio (SNR). These measurements were conducted by a single observer (S.A.) utilizing 0.4-mm-thick axial reformatted images. To determine the signal intensity, the mean CT attenuation (expressed in Hounsfield units [HU]) was measured using circular regions of interest (ROIs; 3–4 mm²) placed within the proximal lumens of both the left and right coronary arteries. Image noise was established as the mean of the standard deviations of CT values across these two ROIs.

The CNR was derived by calculating the difference between the mean attenuation of the proximal coronary arteries and that of the left ventricular lateral wall, then dividing this value by the image noise. Similarly, the SNR was determined by dividing the average signal intensity of the coronary arteries by the image noise.

### Objective image quality analysis of CTAO

Vessel attenuation was measured in six regions of interest, placed in the ascending aorta; aortic arch; descending aorta; abdominal aorta; terminal aorta; common iliac artery. Image noise was defined as the standard deviations of CT attenuation of the psoas muscle. A circular ROI was placed as large as possible in an area where the muscles were uniform at the level of the diaphragm. Then, a circular ROI was placed as large as possible in the aortic lumen. We calculated the difference between the mean CT attenuation values of the aorta and the mean CT attenuation value of the psoas muscle and defined the CNR as the difference divided by image noise. The SNRs were calculated as mean CT attenuation values of the aorta divided by the image noise [11].

### Radiation dose estimation

The volume CT dose index (CTDIvol) and dose-length product (DLP) for each scan were automatically recorded in the dose report generated by the CT scanner. These values were extracted from the participant protocols. The reported total DLP included only CCTA and CTAO acquisitions and excluded the preceding non-contrast coronary calcium scoring scan. For the separate-scan group, total DLP was calculated as the sum of the DLPs from the dedicated CCTA and CTAO acquisitions [12], whereas for the one-scan group, it corresponded to the DLP of the single combined acquisition.

### Statistical analysis

Statistical analysis was performed using R software (version 4.2.2; R Foundation for Statistical Computing, Vienna, Austria) and MedCalc statistical software (version 23.4.8; MedCalc Software Ltd, Ostend, Belgium). Continuous variables were expressed as mean ± standard deviation and categorical variables as number with percentage. Quantitative image quality was compared between separate-scan and one-scan protocol images using the t-test. Subjective CCTA and CTAO image-quality scores were compared between protocols using ordinal generalized estimating equations (GEE) with patient ID specified as the clustering variable and an exchangeable working correlation structure. Odds ratios were interpreted as the odds of receiving a higher image-quality score in the one-scan group compared with the separate-scan group. As a patient-level supportive analysis, segment-level subjective image-quality scores were averaged within each patient, and the resulting patient-level average scores were compared between protocols using the Mann–Whitney U test. Interobserver agreement for the 4-point CCTA and 5-point CTAO image-quality scores was assessed separately for the separate-scan and one-scan groups using quadratic weighted Cohen’s kappa and Gwet’s agreement coefficient 2 (AC2), with quadratic weights. Because multiple coronary segments were evaluated within each patient, 95% confidence intervals (CIs) were estimated using cluster bootstrap resampling at the patient level. A p-value of < 0.05 was considered statistically significant.

## Results

Patient characteristics were comparable between the separate-scan and one-scan protocols (Table 1). The proportion of males was 74% and 78%, respectively. Mean age, body weight, and BMI were similar between the two groups (71 vs. 73 years; 64 vs. 62 kg; 23.6 vs. 22.9 kg/m2). Heart rate at the scan was similar between the groups (60 bpm vs. 61 bpm, *P* = 0.83). Results of coronary artery calcium scores and CCTA are detailed in Supplementary Table 2.

**Table 1:** Patient characteristics

	Separate scan (*n* = 19)	One-scan (*n* = 23)	*P* value
Male, *n* (%)	14 (74)	18 (78)	0.75
Age (y/o)	71 ± 9.6	73 ± 8.7	0.33
Body weight (kg)	63.9 ± 12	62.4 ± 10.1	0.67
BMI (kg/m^2^)	23.6 ± 3.5	22.9 ± 3.5	0.56
Heart rate (bpm)	60 ± 7.3	61 ± 8.6	0.83
Indication for CCTA			
TAA	10	9	
AAA	7	12	
IAA	2	1	
MR	0	1	

Data are presented as the mean ± standard deviation or number of patients (%)

BMI, body mass index; TAA, thoracic aortic aneurysm; AAA, abdominal aortic aneurysm; IAA, iliac arterial aneurysm; MR, mitral regurgitation; CCTA, coronary computed tomography angiography

The subjective and objective image quality assessments of CCTA are summarized in Table 2. In the segment-level subjective visual assessment, CCTA image-quality scores were comparable between the separate-scan and one-scan protocols. In ordinal GEE analysis accounting for within-patient clustering, the one-scan protocol was not associated with significantly different odds of receiving a higher CCTA image-quality score compared with the separate-scan protocol (OR, 1.36; 95% CI, 0.51–3.59; *P* = 0.537). The proportion of coronary segments rated as good or excellent was high in both groups, with 97.1% in the separate-scan group and 98.4% in the one-scan group. Detailed distribution of rated coronary segments is presented in Supplementary Table 3. In the patient-level analysis, the average CCTA image-quality score did not differ significantly between the separate-scan and one-scan groups (3.770 ± 0.320 vs. 3.809 ± 0.284; *P* = 0.918).

**Table 2:** Image quality of CCTA images

	Separate (*n* = 19)	One-scan (*n* = 23)	*P* value
Image quality score			0.537
1. non-diagnostic, n (%)	0 (0)	1 (0.3)	
2. fair, n (%)	8 (2.9)	4 (1.2)	
3. good, n (%)	46 (16.7)	49 (15.0)	
4. excellent, n (%)	221 (80.4)	272 (83.4)	
Patient-level average image-quality score	3.770 ± 0.320	3.809 ± 0.284	0.918
Signal intensity (HU)	842.8 ± 190.1	667.7 ± 179.7	< 0.01
Image noise (HU)	109.3 ± 19.1	110.4 ± 24.1	0.59
Contrast-to-noise ratio	7.0 ± 1.6	4.8 ± 1.9	< 0.01
Signal-to-noise ratio	8.2 ± 1.8	6.3 ± 2.0	< 0.01

Data are presented as the mean ± standard deviation or number of patients (%). Subjective image quality scores are presented per coronary segment; objective parameters are presented per patient

CCTA, Coronary computed tomography angiography; HU, Hounsfield unit

**Fig. 2:**
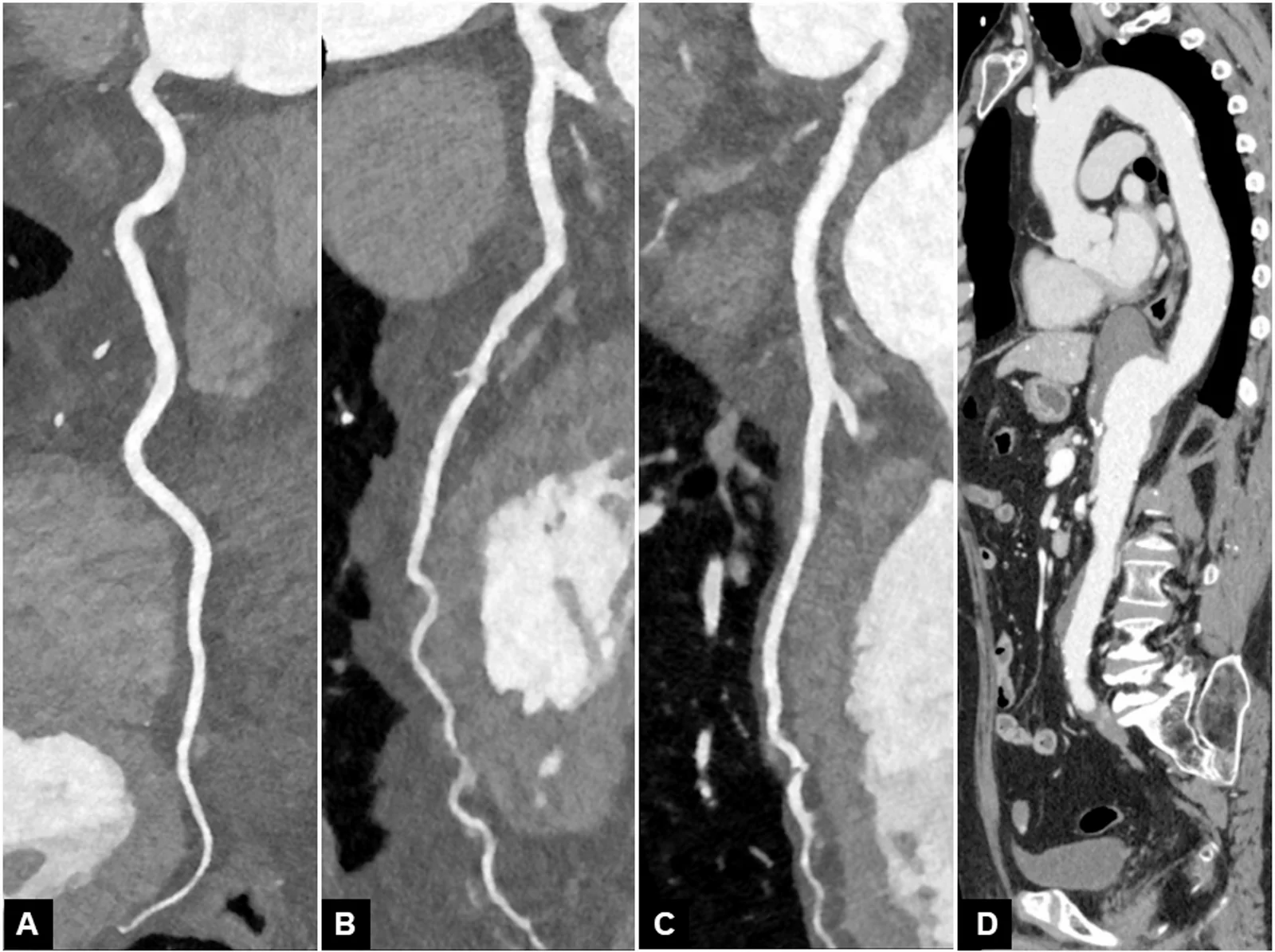
Representative case in the separate-scan group. A patient in their 70s with an average body habitus underwent preoperative CCTA and CTAO for thoracoabdominal aortic aneurysm. The patient underwent the separate-scan protocol because dedicated coronary assessment was required in addition to preoperative aortic evaluation. The heart rate during scanning was 58 beats/min. Figure **A**-**C** show the right coronary artery, left anterior descending artery, and left circumflex artery on CCTA, respectively, and Figure **D** shows CTAO. This case illustrates the coronary-optimized nature of the separate-scan protocol, which was associated with higher coronary attenuation and CNR at the group level, while presenting excellent visual coronary image quality. Aortic image quality was also rated as excellent. Display settings were as follows: CCTA, window level 400 and window width 2500; CTAO, window level 40 and window width 400. Window settings were individually optimized for each representative case to provide the clearest visualization; therefore, these figures were not intended for direct visual comparison between protocols under identical display settings. CCTA, coronary computed tomography angiography; CTAO, computed tomography angiography of the aorta

Regarding objective image quality parameters, signal intensity was significantly higher in the separate-scan group compared to the one-scan group (842.8 ± 190.1 HU vs. 667.7 ± 179.7 HU; *P* < 0.01). However, there was no significant difference in image noise between the two protocols (109.3 ± 19.1 HU vs. 110.4 ± 24.1 HU; *P* = 0.59). Consequently, the separate-scan group demonstrated significantly higher CNR (7.0 ± 1.6 vs. 4.8 ± 1.9; *P* < 0.01) and SNR (8.2 ± 1.8 vs. 6.3 ± 2.0; *P* < 0.01) compared to the one-scan group (Fig. 2).

The subjective and objective image quality assessments of CTAO are summarized in Table 3. In the segment-level subjective visual assessment, CTAO image-quality scores were significantly higher in the one-scan protocol than in the separate-scan protocol. In ordinal GEE analysis accounting for within-patient clustering with an exchangeable working correlation structure, the one-scan protocol was associated with significantly higher odds of receiving a better CTAO image-quality score compared with the separate-scan protocol (OR, 2.52; 95% CI, 1.12–5.65; *P* = 0.025). Image quality scores of the aorta were predominantly clustered between 4 and 5 across the evaluated aortic segments (Supplementary Table 4). The proportion of aortic segments rated as good or excellent was 91.3% in the one-scan group, compared to 85.1% in the separate-scan group. In the patient-level supportive analysis, the average CTAO image-quality score was also significantly higher in the one-scan group than in the separate-scan group (4.652 ± 0.411 vs. 4.412 ± 0.453; *P* = 0.048).

**Table 3:** Image quality of CT aortography images

	Separate scan (*n* = 19)	One-scan (*n* = 23)	*P* value
Image quality score			0.025
1. non-diagnostic, n (%)	0 (0)	0 (0)	
2. poor, n (%)	0 (0)	1 (0.7)	
3. moderate, n (%)	17 (14.9)	11 (8.0)	
4. good, n (%)	33 (28.9)	23 (16.7)	
5. excellent, n (%)	64 (56.1)	103 (74.6)	
Patient-level average image-quality score	4.412 ± 0.453	4.652 ± 0.411	0.048
Signal intensity (HU)	379.1 ± 134.2	484.9 ± 121.1	0.01
Image noise (HU)	13.2 ± 3.1	21.2 ± 5.3	< 0.01
Contrast-to-noise ratio	26.7 ± 15.4	21.2 ± 7.2	0.13
Signal-to-noise ratio	31.3 ± 16.3	23.8 ± 7.6	0.06

Data are presented as the mean ± standard deviation or number of patients (%). Subjective image quality scores are presented per aortic segment; objective parameters are presented per patient

CT, computed tomography; HU, Hounsfield unit

**Fig. 3:**
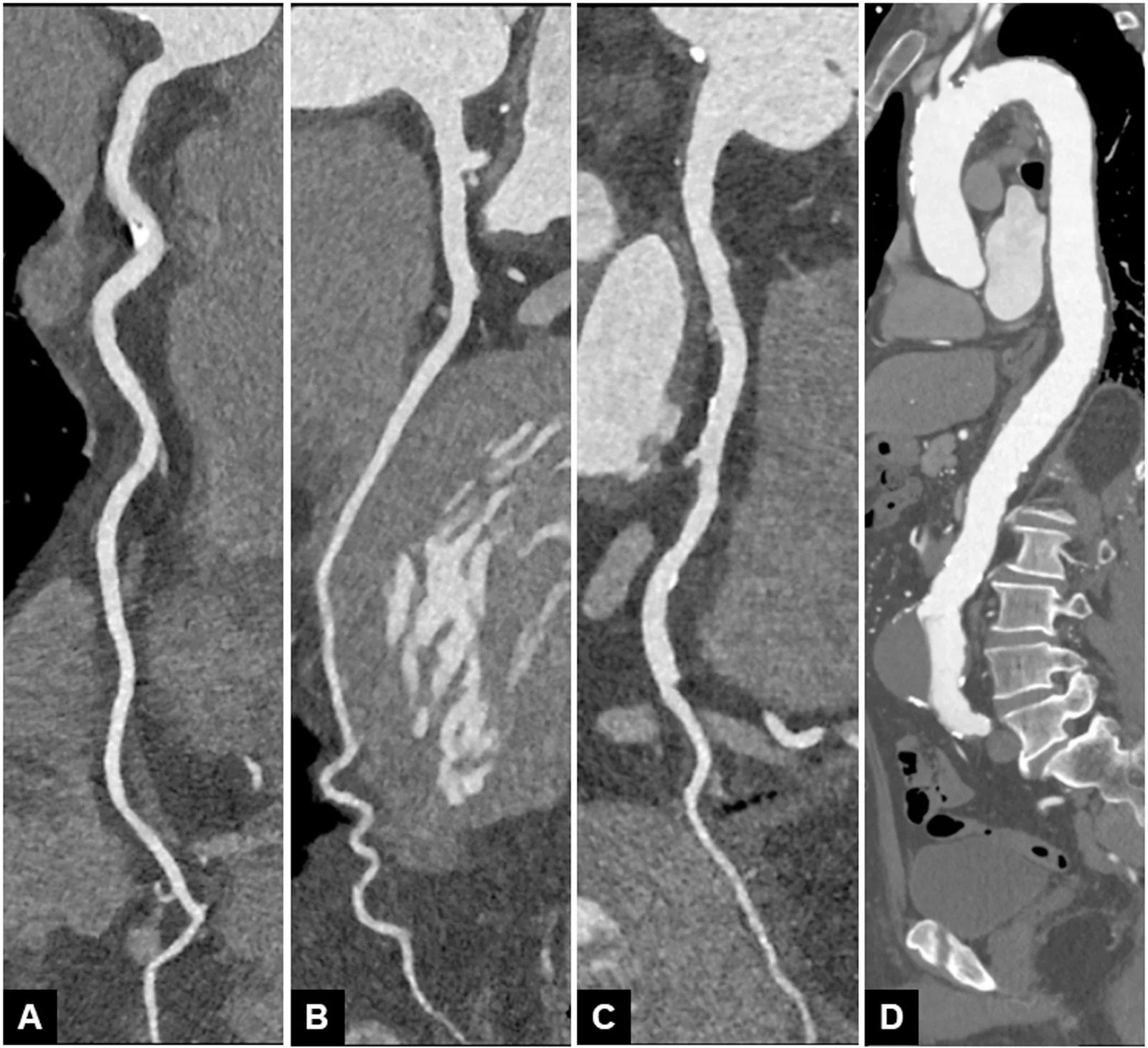
Representative case in the one-scan group. A patient in their 70s with an average body habitus underwent preoperative CCTA and CTAO for abdominal aortic aneurysm. The patient underwent the one-scan protocol because comprehensive aortic assessment, including evaluation of the access route, was the primary clinical objective, and concomitant coronary assessment was obtained in the same acquisition. The heart rate during scanning was 50 beats/min. Figure **A**-**C** show the right coronary artery, left anterior descending artery, and left circumflex artery on CCTA, respectively, and Figure **D** shows CTAO. This case illustrates the aorta-oriented timing of the one-scan protocol, which was associated with higher aortic attenuation at the group level despite higher image noise. Both coronary and aortic image quality were rated as excellent. Display settings were as follows: CCTA, window level 400 and window width 1500; CTAO, window level 200 and window width 1200. Window settings were individually optimized for each representative case to provide the clearest visualization; therefore, these figures were not intended for direct visual comparison between protocols under identical display settings. CCTA, coronary computed tomography angiography; CTAO, computed tomography angiography of the aorta

Regarding objective image quality parameters, the one-scan group demonstrated significantly higher signal intensity within the aorta compared to the separate-scan group (484.9 ± 121.1 HU vs. 379.1 ± 134.2 HU; *P* = 0.01). However, image noise was also significantly higher in the one-scan group (21.2 ± 5.3 HU) compared to the separate-scan group (13.2 ± 3.1 HU; *P* < 0.01). Consequently, the SNR showed a trend toward being lower in the one-scan group compared to the separate-scan group (23.8 ± 7.6 vs. 31.3 ± 16.3; *P* = 0.06). The CNR also showed a trend toward being lower in the one-scan group (21.2 ± 7.2 vs. 26.7 ± 15.4; *P* = 0.13) (Fig. 3).

Interobserver agreement for subjective CCTA image quality was high in both protocols (Supplementary Table 5). In the separate-scan group, complete agreement was observed in 92.4% of 275 paired evaluations, with a quadratic weighted κ of 0.847 (95% CI, 0.692–0.917) and Gwet’s AC2 of 0.987 (95% CI, 0.982–0.993). In the one-scan group, complete agreement was 92.6% among 326 paired evaluations, with a quadratic weighted κ of 0.816 (95% CI, 0.648–0.903) and Gwet’s AC2 of 0.989 (95% CI, 0.984–0.994). For subjective CTAO image quality, complete agreement was 59.6% among 114 paired evaluations in the separate-scan group and 77.5% among 138 paired evaluations in the one-scan group. The quadratic weighted κ values were 0.667 (95% CI, 0.502–0.784) and 0.552 (95% CI, 0.353–0.759), respectively, whereas Gwet’s AC2 values were 0.971 (95% CI, 0.950–0.987) and 0.975 (95% CI, 0.952–0.991), respectively.

**Fig. 4:**
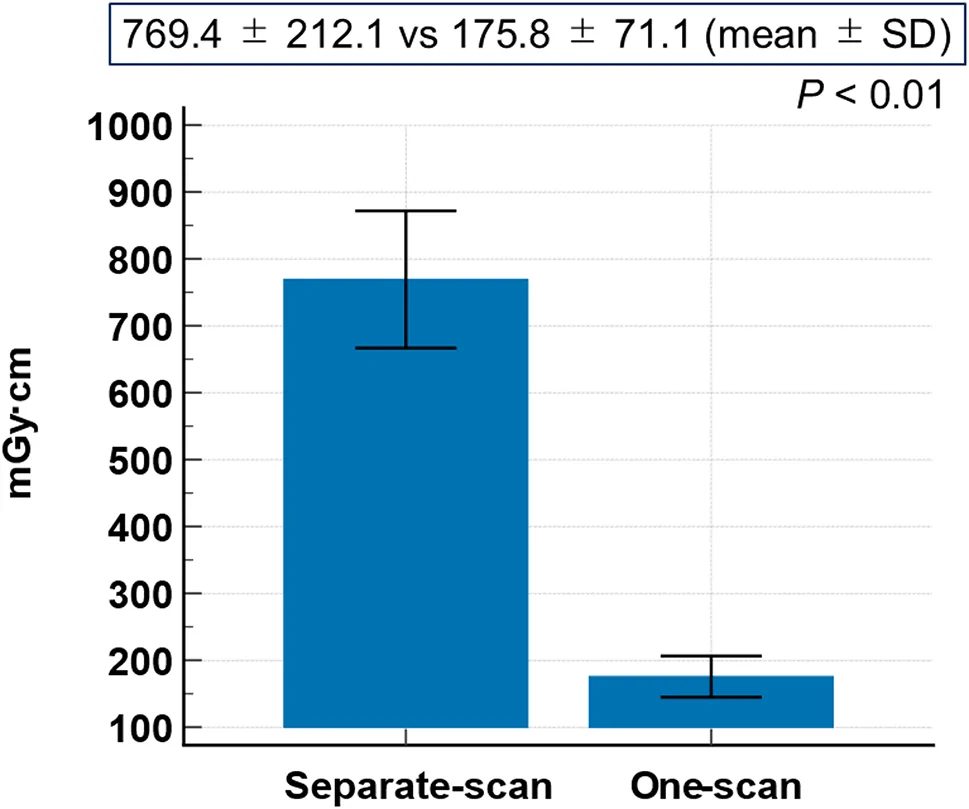
Comparison of radiation dose between the separate scan and one-scan group. The mean total dose-length product was significantly lower in the one-scan group compared to the separate scan group (175.8 ± 71.1 mGy·cm vs. 769.4 ± 212.1 mGy·cm; P < 0.01). Error bars represent standard deviations

The mean DLP was significantly lower with the one-scan protocol compared to the separate-scan protocol (separate scan vs. one-scan; 769.4 ± 212.1 vs. 175.8 ± 71.1 mGy·cm; *P* < 0.01) (Fig. 4). In the separate-scan group, the mean DLPs of CCTA and CTAO were 116.1 ± 121.1 and 673.3 ± 177.8 mGy·cm, respectively. The mean CTDIvol of one-scan protocol was 3.2 ± 2.6 mGy, whereas the mean CTDIvol of CCTA and CTAO were 9.5 ± 10.9 and 9.6 ± 2.5 mGy, respectively in the separate-scan group.

## Discussion

### Principal findings

The present study compared two examination strategies on dual-source photon-counting detector CT (PCD-CT): a low-kV, ECG-gated high-pitch one-scan protocol designed primarily for thoracoabdominal CTAO while simultaneously including coronary imaging, and a conventional strategy consisting of dedicated CCTA plus separate standard-helical CTAO at higher tube voltage. Three findings merit emphasis. First, the one-scan strategy markedly reduced radiation dose. Second, although objective coronary contrast metrics were lower with the one-scan protocol, subjective CCTA image-quality scores did not differ significantly between the two strategies. Third, subjective aortic image quality was superior with the one-scan strategy despite lower objective aortic CNR.

### Comparison with previous studies of high-pitch thoracoabdominal CTAO

Published adult PCD-CT studies of ECG-gated high-pitch thoracoabdominal CTAO have generally been designed as dedicated aortic examinations under standard tube-voltage conditions rather than as combined aortic-coronary protocols. In the available literature, Hennes et al. [13] used a descending thoracic aortic ROI with a 100-HU trigger and a fixed 7-s delay, whereas Rippel et al. (2025) [4], Rippel et al. (2024) [14], Dillinger et al. [15], and Yang et al. [16] all used proximal aortic triggering, typically in the ascending aorta with a 100-HU threshold, followed by protocol-specific fixed delays or sequential acquisition steps (Table 4). Importantly, these adult PCD-CT high-pitch thoracoabdominal CTAO studies were performed at 120 kVp, did not formally incorporate concomitant CCTA within the same examination as a study objective, and did not evaluate the image quality of coronary assessment obtained during the same acquisition. In contrast, the present study used a low-kV (70/90 kVp), ECG-gated high-pitch one-scan protocol in which the timing reference was intentionally moved downstream to the iliac crest with a 150-HU trigger and a fixed 5-s delay. Thus, our protocol was specifically designed to favor distal aortoiliac opacification while simultaneously testing whether coronary assessment obtained under such conditions would remain clinically acceptable compared with dedicated CCTA.

**Table 4:** Comparison of adult PCD-CT high-pitch helical CTAO studies and the present study, including concomitant CCTA acquisition and coronary image-quality evaluation

Study	Research purpose	ECG synchronization	Tube voltage	Radiation dose (reported metric)	Concomitant CCTA acquisition within the same examination	Image-quality evaluation of concomitant CCTA	Bolus-tracking ROI / trigger scheme
Hennes et al., 2023	Intra-individual comparison of PCD-CT vs. EID-CT for CTAO	Yes (ECG-gated)	120 kVp	CTDIvol 3.95 ± 0.54 mGy	No	No	Descending thoracic aorta ROI; 100 HU trigger; fixed 7-s delay
Rippel et al., 2025	Pre-TAVR CTAO: dose/image-quality evaluation and VMI utility	Yes (ECG-gated)	120 kVp	CTDIvol 4.0 [3.1–4.9] mGy; DLP 288 [207–402] mGy·cm	No	No	Ascending aorta ROI; 100 HU trigger; fixed 11-s delay
Rippel et al., 2024	Pre-TAVR CTAO: low-keV VMI to salvage suboptimal iliofemoral enhancement	Yes (ECG-gated)	120 kVp	CTDIvol 4.1 ± 1.0 mGy; DLP 473 ± 172.5 mGy·cm	No	No	Ascending aorta ROI; 100 HU trigger; 5-s delay
Yang et al., 2024	Pre-TAVR: annulus sizing accuracy with prospective ECG-gated high-pitch acquisition and access-route visualization	Yes (ECG-gated; the aortic valve level targeted at 30% R-R)	120 kVp	CTDIvol 4.5 ± 1.13 mGy; DLP 317.0 ± 92.26 mGy·cm†	No	No	Ascending aorta ROI; 100 HU trigger; cardiac spiral-CT followed immediately by high-pitch CTAO
Dillinger et al., 2025	Dedicated aortic dissection protocol; evaluation of VME/VNC-based imaging	Yes (ECG-gated)	120 kVp	CTDIvol 5.02 ± 1.77 mGy; DLP 386.71 ± 144.97 mGy·cm‡	No	No	Ascending aorta ROI; 100 HU trigger
The present study	Low-kV ECG-triggered high-pitch one-scan CCTA+CTAO vs. separate dedicated CCTA plus standard-helical CTAO	Yes (ECG-gated; CCTA targeted at 70% R-R)	One-scan: 70/90 kVp	One-scan: CTDIvol 3.2 ± 2.6 mGy; Total DLP 175.8 ± 71.1 mGy·cm	Yes - one-scan simultaneous CCTA+CTAO (Turbo Flash Chest Pain)	Yes - subjective and objective coronary image-quality comparison vs. dedicated CCTA	ROI at iliac-crest level; 150 HU trigger; fixed 5-s delay

Abbreviations: CCTA, coronary computed tomography angiography; CTAO, CT angiography of the aorta; CTDI, CT dose index; CTDIvol,volume CT dose index; DLP, dose-length product; ECG, electrocardiographic; EID-CT, energy-integrating detector CT; PCD-CT, photon-counting detector CT; ROI, region of interest; SSDE, size-specific dose estimate; TAVR, transcatheter aortic valve replacement; VME, virtualmonoenergetic enhancement; VMI, virtual monoenergetic imaging; VNC, virtual non-contrast

### Radiation dose reduction was driven primarily by the CTAO acquisition strategy

The substantial dose reduction achieved by the one-scan strategy appears to be attributable primarily to the CTAO acquisition strategy rather than merely to the elimination of overlapping scan ranges. In the separate-acquisition group, the CTAO component accounted for most of the total DLP, indicating that thoracoabdominal aortic imaging was the principal determinant of overall radiation exposure. By contrast, the one-scan protocol yielded a total DLP of 175.8 ± 71.1 mGy·cm despite simultaneously including coronary imaging. Although cross-study comparison should be interpreted cautiously because prior adult PCD-CT high-pitch thoracoabdominal CTAO studies generally reported CTAO-specific CTDIvol or DLP rather than total examination DLP, the dose level of our low-kV one-scan protocol was lower than the reported DLP values of previously published adult 120-kVp ECG-gated high-pitch PCD-CT CTAO studies, including 288 [207–402] mGy·cm in Rippel et al., 317.0 ± 92.26 mGy·cm in Yang et al., 386.71 ± 144.97 mGy·cm in Dillinger et al., and 473 ± 172.5 mGy·cm in Rippel et al. [14–16]. These findings suggest that the marked reduction in total radiation dose in the present study was driven predominantly by replacing conventional higher-kV CTAO with a low-kV ECG-gated high-pitch thoracoabdominal acquisition, while simultaneously avoiding an additional CCTA scan.

### High-pitch CCTA in a one-scan protocol

Prospectively ECG-triggered high-pitch helical acquisition is an established scan technique for CCTA on dual-source CT, and previous studies have demonstrated a marked reduction in radiation exposure while preserving acceptable image quality. For example, Schicchi et al. [17] demonstrated that prospective high-pitch turbo-flash CCTA substantially reduced measured and estimated radiation doses compared with retrospective ECG-gated dual-source CT, without a significant difference in subjective image quality. The present one-scan protocol applied this dose-saving principle to a substantially longer scan range by incorporating coronary and thoracoabdominal aortic imaging into a single acquisition.

Assessing the coronary arteries within a single ECG-triggered high-pitch acquisition performed primarily for another purpose is not a new concept. Fogante et al. [18] reported an ultra-low-radiation-dose and low-contrast-volume high-pitch protocol for TAVI planning that combined evaluation of the aortic valve, access route, coronary arteries, and calcium burden. Although its clinical indication and protocol design differed from those of the present study, their findings further illustrate the potential value of integrating multiple cardiovascular assessments into a high-pitch protocol. However, unlike the TAVI study, we did not evaluate coronary diagnostic accuracy against invasive coronary angiography, and the adequacy of our protocol for CAD screening remains to be established.

### Timing trade-off and coronary image quality

While ECG-gating at the cardiac quiescent phase (e.g., 70% of the R-R interval in our protocol) effectively minimizes coronary motion artifacts, achieving simultaneous peak contrast enhancement for both the coronary arteries and the entire thoracoabdominal aorta within a single, rapid acquisition is challenging. Prior EID-CT studies have explored the feasibility of concomitant coronary assessment, reporting variable diagnostic rates (e.g., 97.2% of segments diagnostic in Goetti et al. vs. 47.6% in Scharf et al.) [19, 20]. Given this context, our study uniquely provides a direct comparison of concomitant coronary assessment from an aorta-oriented one-scan protocol with dedicated CCTA. In our study, coronary attenuation, CNR, and SNR were lower in the one-scan group, whereas image noise was similar between groups. Importantly, however, subjective CCTA image-quality scores did not differ significantly, and the proportion of segments rated as good or excellent remained very high in both groups. These findings suggest that, although the timing trade-off was not fully overcome, concomitant coronary assessment in the one-scan protocol remained clinically acceptable for preoperative CAD screening in appropriately selected patients.

### CTAO timing design and aortic image quality

From the standpoint of CTAO, only the one-scan protocol used an aorta-oriented timing scheme, while CTAO in the separate-acquisition strategy was obtained as a delayed follow-on scan after coronary-optimized CCTA. The former was designed to favor distal aortoiliac enhancement, whereas the latter likely rendered CTAO relatively late and potentially suboptimal for homogeneous thoracoabdominal and distal aortoiliac opacification. This interpretation is consistent with our results: the one-scan group showed significantly higher aortic attenuation, but also higher image noise, resulting in lower calculated CNR. Importantly, however, readers rated subjective CTAO image quality as superior in the one-scan group. This discrepancy suggests that the visual ratings may have been influenced mainly by stronger luminal enhancement, which facilitated delineation of the aortic lumen despite increased noise. Subjective assessment may also reflect visual characteristics not fully captured by CNR and SNR alone. Therefore, the higher subjective scores should not be interpreted as indicating overall superiority of CTAO image quality. Rather, the one-scan protocol provided a different balance between luminal attenuation and image noise, producing a favorable visual impression without superior objective image-quality metrics.

However, because the two protocols employed different contrast timing strategies, the superior subjective CTAO image quality observed with the one-scan protocol should not be attributed solely to the one-scan acquisition itself or to PCD-CT performance. Rather, differences in contrast enhancement timing likely contributed, at least in part, to the observed advantage.

### The role of PCD-CT in a low-kV high-pitch strategy

The feasibility of this strategy is likely supported by the detector physics of PCD-CT, in which each incoming photon is converted directly into an electrical pulse within a semiconductor detector and is counted only if the pulse exceeds a preset energy threshold, thereby reducing the contribution of electronic noise, especially at low photon counts. This direct-conversion, threshold-based design improves dose efficiency and helps preserve image quality under low-kV, high-pitch conditions. In this context, the role of PCD-CT is not that it eliminates the timing trade-off itself, but rather that it makes an aorta-oriented low-dose acquisition clinically usable despite that trade-off. The present study therefore extends the concept of low-dose high-pitch cardiovascular CT, previously demonstrated for dedicated CCTA, to combined thoracoabdominal aortic imaging, where the potential for dose reduction is even greater because of the long scan range. Importantly, this advantage is likely relevant not only to CTAO but also to concomitant CCTA in the one-scan protocol. However, the present study did not include an EID-CT control group and was not designed to isolate the independent effect of detector technology. The feasibility of the one-scan strategy should not be attributed solely to the performance of PCD-CT based on the present findings. Direct comparative studies using otherwise matched PCD-CT and EID-CT protocols are needed to determine the specific contribution of PCD-CT.

### Clinical implications

These findings have practical implications for preoperative imaging workflows. When comprehensive evaluation of the thoracoabdominal aorta and access route is the primary imaging objective, a low-kV high-pitch one-scan strategy may provide substantial dose savings while simultaneously yielding coronary information sufficient for screening purposes. Conversely, when detailed coronary characterization is the primary clinical question, a dedicated CCTA-oriented protocol may remain preferable. Thus, the present results do not suggest that one-scan imaging should replace separate acquisitions in all circumstances; rather, they support its use as a dose-efficient alternative when aortic evaluation is the dominant objective and concomitant coronary screening is also desired.

## Limitations

This study has several limitations. First, it was a single-center retrospective study with a relatively small sample size, and protocol selection was based on the primary clinical objective rather than randomization; therefore, selection bias and limited generalizability cannot be excluded. Second, we compared two overall examination strategies, so the observed differences reflect the combined effects of multiple factors, including tube voltage, pitch, ECG synchronization, contrast timing, and simultaneous versus separate acquisition, rather than the isolated effect of a single parameter. In particular, the superior subjective CTAO image quality observed with the one-scan protocol may have been influenced, at least in part, by differences in contrast timing. Third, although subjective coronary image quality in the one-scan protocol was favorable, diagnostic accuracy for coronary stenosis was not evaluated against a reference standard. Therefore, our findings should not be interpreted as demonstrating diagnostic equivalence for coronary artery disease detection. The adequacy of the one-scan protocol for preoperative CAD screening remains uncertain. Fourth, our population had relatively well-controlled heart rates and a relatively low body mass index. In addition, patients with prior coronary stent placement, prior aortic surgery, double-barrel aortic dissection, or significant metal artifacts were excluded, and the effect of severe coronary calcification was not specifically evaluated. Therefore, the performance of the one-scan protocol in patients with obesity, tachycardia, arrhythmia, severe coronary calcification, or complex postoperative anatomy remains uncertain. Finally, we did not assess the incremental contribution of specific spectral reconstructions or evaluate downstream clinical outcomes, including subsequent coronary testing, invasive coronary angiography, or changes in clinical management.

## Conclusion

On dual-source photon-counting detector CT, a low-kV ECG-gated high-pitch one-scan strategy primarily designed for thoracoabdominal CTAO substantially reduced radiation dose compared with a conventional strategy consisting of dedicated CCTA plus separate standard-helical CTAO at higher tube voltage. Although objective coronary contrast metrics were lower, concomitant coronary assessment remained subjectively comparable to dedicated CCTA, and subjective aortic image quality was superior. These findings support the feasibility of the one-scan approach as a practical low-dose option when aortic assessment is the primary imaging objective and coronary screening is also required. However, its diagnostic sufficiency for coronary stenosis was not established and further studies using an appropriate reference standard are needed.

## Supplementary Information

Below is the link to the electronic supplementary material.

Supplementary Material 1

Supplementary Material 2

## Data Availability

The datasets used and/or analyzed during the current study are available from the corresponding author on reasonable request.

## Abbreviations

CAD: Coronary artery disease
CCTA: Coronary computed tomography angiography
CTAO: CT aortography
PCD-CT: Photon-counting detector CT
EID: Energy-integrating detector
ECG: Electrocardiogram
bpm: beats per minute
heart rate: HR
QIR: Quantum iterative reconstruction
CNR: Contrast-to-noise ratio
SNR: Signal-to-noise ratio
HU: Hounsfield unit
DLP: Dose-length product
