# Supplementary material for: Low-kV high-pitch one-scan aortic and coronary CT angiography using dual-source photon-counting CT: comparison with separate acquisition strategies
Source: BMC Med Imaging. 2026 Jul 31;26:573. doi: 10.1186/s12880-026-02629-0 (PMC13729364; doi:10.1186/s12880-026-02629-0)
Supplement: Supplementary file 1 [file 12880_2026_2629_MOESM1_ESM.docx]

**Supplemental material**

**Supplemental Figure 1. Illustration of image quality scores of coronary arteries on CCTA images.**

The figures show a 4-point scale used to assess the image quality of the CCTA images: non-diagnostic; (2) fair; (3) good; and (4) excellent. In score 2 (fair) image, luminal evaluation is limited due to calcification (arrow). CCTA, coronary computed tomography angiography.

**Supplemental Figure 2. Illustration of image quality scores of coronary arteries on CTAO images.**

The figures show a 5-point scale used to assess the image quality of the CTAO images: (1) non-diagnostic; (2) fair; (3) moderate; (4) good; and (5) excellent. The score 4 (good) image shows good contrast enhancement in the descending aorta (arrow), whereas the ascending aorta demonstrates relatively weaker enhancement and was graded as score 3 (moderate). CTAO, computed tomography aortography.

**Supplementary Table 1. Comparison of CT acquisition parameters between the separate scan and one scan protocols**

|  | Separate scan (n=19) | One-scan (n=23) |
| --- | --- | --- |
| β-blocker use | 19 (100) | 23 (100) |
| Tube voltage (kVp) |  |  |
| CCTA |  |  |
| 70 | 11 (58) | NA |
| 90 | 4 (21) | NA |
| 120 | 4 (21) | NA |
| CTAO |  |  |
| 70 | 0 (0) | 7 (30) |
| 90 | 0 (0) | 16 (70) |
| 120 | 13 (68) | 0 (0) |
| 140 | 6 (32) | 0 (0) |
| CCTA acquisition mode |  |  |
| Sequential | 8 (42) | 0 (0) |
| Flash spiral | 11 (58) | 23 (100) |
| Scan length (cm) |  |  |
| CCTA | 14.7 ± 3.1 | NA |
| CTAO | 67.8 ± 8.0 | 67.6 ± 3.7 |
| Contrast volume (ml) | 55.7 ± 7.3 | 61.7 ± 14.7 |

Data are presented as the mean ± standard deviation or number of patients (%).

CCTA, coronary computed tomography angiography; CTAO, computed tomography aortography; NA, not applicable.

**Supplementary Table 2. Comparison of baseline coronary artery disease burden between the separate scan and the one-scan groups**

|  | Separate scan (n=19) | One-scan (n=23) | *P* value |
| --- | --- | --- | --- |
| CACS, n (%) CACS, median (interquartile range) | 313 (38–908) | 171 (37–1039) | 0.64 |
| CACS category, n (%) |  |  |  |
| CACS 0 | 1 (5) | 4 (17) |  |
| CACS 1-99 | 6 (32) | 5 (22) |  |
| CACS 100-399 | 4 (21) | 4 (17) |  |
| CACS > 400 | 8 (42) | 10 (44) |  |
| Obstructive CAD on CCTA, n (%) | 8 (42) | 12 (52) | 0.52 |
| CAD-RADS |  |  |  |
| CAD-RADS 0 | 1 (5) | 3 (13) |  |
| CAD-RADS 1 | 6 (32) | 3 (13) |  |
| CAD-RADS 2 | 4 (21) | 5 (22) |  |
| CAD-RADS 3 | 0 (0) | 2 (9) |  |
| CAD-RADS 4A | 4 (21) | 6 (26) |  |
| CAD-RADS 4B | 3 (16) | 2 (9) |  |
| CAD-RADS 5 | 1 (5) | 2 (9) |  |

Data are presented as the mean ± standard deviation or number of patients (%). Obstructive CAD was defined as CAD-RADS ≥3

CACS, coronary artery calcium score; CAD, coronary artery disease; CAD-RADS, coronary artery disease reporting and data system; CCTA, coronary computed tomography angiography.
