# Supplementary material for: Low-kV high-pitch one-scan aortic and coronary CT angiography using dual-source photon-counting CT: comparison with separate acquisition strategies
Source: BMC Med Imaging. 2026 Jul 31;26:573. doi: 10.1186/s12880-026-02629-0 (PMC13729364; doi:10.1186/s12880-026-02629-0)
Supplement: Supplementary file 2 [file 12880_2026_2629_MOESM2_ESM.docx]

**Supplemental Table 3. Segment-specific distribution of subjective CCTA image-quality scores**

| **Coronary segment** | **Group** | **Evaluated, n** | **Image-quality score, n (%)** | | | | |
| --- | --- | --- | --- | --- | --- | --- | --- |
|  |  |  | **1: Non-diagnostic** | **2: Fair** | **3: Good** | **4: Excellent** | **3-4: Good/excellent** |
| Proximal RCA | Separate-scan | 19 | 0 (0.0) | 1 (5.3) | 3 (15.8) | 15 (78.9) | 18 (94.7) |
|  | One-scan | 23 | 0 (0.0) | 1 (4.3) | 3 (13.0) | 19 (82.6) | 22 (95.7) |
| Mid RCA | Separate-scan | 19 | 0 (0.0) | 1 (5.3) | 3 (15.8) | 15 (78.9) | 18 (94.7) |
|  | One-scan | 22 | 0 (0.0) | 1 (4.5) | 7 (31.8) | 14 (63.6) | 21 (95.5) |
| Distal RCA | Separate-scan | 18 | 0 (0.0) | 1 (5.6) | 2 (11.1) | 15 (83.3) | 17 (94.4) |
|  | One-scan | 21 | 0 (0.0) | 1 (4.8) | 3 (14.3) | 17 (81.0) | 20 (95.2) |
| RCA-PDA | Separate-scan | 17 | 0 (0.0) | 0 (0.0) | 3 (17.6) | 14 (82.4) | 17 (100.0) |
|  | One-scan | 21 | 0 (0.0) | 0 (0.0) | 4 (19.0) | 17 (81.0) | 21 (100.0) |
| RCA-PLB | Separate-scan | 17 | 0 (0.0) | 0 (0.0) | 4 (23.5) | 13 (76.5) | 17 (100.0) |
|  | One-scan | 21 | 0 (0.0) | 0 (0.0) | 3 (14.3) | 18 (85.7) | 21 (100.0) |
| LMCA | Separate-scan | 19 | 0 (0.0) | 0 (0.0) | 1 (5.3) | 18 (94.7) | 19 (100.0) |
|  | One-scan | 23 | 0 (0.0) | 0 (0.0) | 0 (0.0) | 23 (100.0) | 23 (100.0) |
| Proximal LAD | Separate-scan | 19 | 0 (0.0) | 0 (0.0) | 3 (15.8) | 16 (84.2) | 19 (100.0) |
|  | One-scan | 23 | 0 (0.0) | 0 (0.0) | 2 (8.7) | 21 (91.3) | 23 (100.0) |
| Mid LAD | Separate-scan | 19 | 0 (0.0) | 0 (0.0) | 3 (15.8) | 16 (84.2) | 19 (100.0) |
|  | One-scan | 23 | 0 (0.0) | 0 (0.0) | 3 (13.0) | 20 (87.0) | 23 (100.0) |
| Distal LAD | Separate-scan | 19 | 0 (0.0) | 2 (10.5) | 3 (15.8) | 14 (73.7) | 17 (89.5) |
|  | One-scan | 23 | 0 (0.0) | 0 (0.0) | 4 (17.4) | 19 (82.6) | 23 (100.0) |
| First diagonal branch | Separate-scan | 19 | 0 (0.0) | 1 (5.3) | 2 (10.5) | 16 (84.2) | 18 (94.7) |
|  | One-scan | 23 | 0 (0.0) | 0 (0.0) | 5 (21.7) | 18 (78.3) | 23 (100.0) |
| Second diagonal branch | Separate-scan | 18 | 0 (0.0) | 0 (0.0) | 4 (22.2) | 14 (77.8) | 18 (100.0) |
|  | One-scan | 14 | 0 (0.0) | 0 (0.0) | 2 (14.3) | 12 (85.7) | 14 (100.0) |
| Proximal LCX | Separate-scan | 19 | 0 (0.0) | 0 (0.0) | 3 (15.8) | 16 (84.2) | 19 (100.0) |
|  | One-scan | 23 | 0 (0.0) | 0 (0.0) | 2 (8.7) | 21 (91.3) | 23 (100.0) |
| First obtuse marginal branch | Separate-scan | 18 | 0 (0.0) | 0 (0.0) | 4 (22.2) | 14 (77.8) | 18 (100.0) |
|  | One-scan | 22 | 0 (0.0) | 1 (4.5) | 4 (18.2) | 17 (77.3) | 21 (95.5) |
| Distal LCX | Separate-scan | 18 | 0 (0.0) | 2 (11.1) | 2 (11.1) | 14 (77.8) | 16 (88.9) |
|  | One-scan | 22 | 1 (4.5) | 0 (0.0) | 4 (18.2) | 17 (77.3) | 21 (95.5) |
| Second obtuse marginal branch | Separate-scan | 8 | 0 (0.0) | 0 (0.0) | 0 (0.0) | 8 (100.0) | 8 (100.0) |
|  | One-scan | 9 | 0 (0.0) | 0 (0.0) | 0 (0.0) | 9 (100.0) | 9 (100.0) |
| LCX-PDA | Separate-scan | 2 | 0 (0.0) | 0 (0.0) | 1 (50.0) | 1 (50.0) | 2 (100.0) |
|  | One-scan | 2 | 0 (0.0) | 0 (0.0) | 0 (0.0) | 2 (100.0) | 2 (100.0) |
| LCX-PLB | Separate-scan | 1 | 0 (0.0) | 0 (0.0) | 1 (100.0) | 0 (0.0) | 1 (100.0) |
|  | One-scan | 2 | 0 (0.0) | 0 (0.0) | 1 (50.0) | 1 (50.0) | 2 (100.0) |
| Ramus intermedius | Separate-scan | 6 | 0 (0.0) | 0 (0.0) | 4 (66.7) | 2 (33.3) | 6 (100.0) |
|  | One-scan | 9 | 0 (0.0) | 0 (0.0) | 2 (22.2) | 7 (77.8) | 9 (100.0) |
| Overall | Separate-scan | 275 | 0 (0.0) | 8 (2.9) | 46 (16.7) | 221 (80.4) | 267 (97.1) |
|  | One-scan | 326 | 1 (0.3) | 4 (1.2) | 49 (15.0) | 272 (83.4) | 321 (98.5) |

Data are numbers of evaluated coronary segments, with percentages.

CCTA, coronary computed tomography angiography; LAD, left anterior descending artery; LCX, left circumflex artery; LMCA, left main coronary artery; OM, obtuse marginal branch; PDA, posterior descending artery; PLB, posterolateral branch; RCA, right coronary artery.

**Supplemental Table 4. Segment-specific distribution of subjective CTAO image-quality scores**

| **Aortic segment** | **Separate scan (n = 19)** | | | | | | | **One-scan (n = 23)** | | | | | | |
| --- | --- | --- | --- | --- | --- | --- | --- | --- | --- | --- | --- | --- | --- | --- |
|  | **Mean ± SD** | **Score 1 n (%)** | **Score 2 n (%)** | **Score 3 n (%)** | **Score 4 n (%)** | **Score 5 n (%)** | **Good/excellent (scores 4–5), n (%)** | **Mean ± SD** | **Score 1 n (%)** | **Score 2 n (%)** | **Score 3 n (%)** | **Score 4 n (%)** | **Score 5 n (%)** | **Good/excellent (scores 4–5), n (%)** |
| Ascending aorta | 4.3 ± 0.6 | 0 (0.0) | 0 (0.0) | 1 (5.3) | 11 (57.9) | 7 (36.8) | 18 (94.7) | 4.5 ± 0.7 | 0 (0.0) | 0 (0.0) | 2 (8.7) | 8 (34.8) | 13 (56.5) | 21 (91.3) |
| Aortic arch | 4.5 ± 0.6 | 0 (0.0) | 0 (0.0) | 1 (5.3) | 7 (36.8) | 11 (57.9) | 18 (94.7) | 4.7 ± 0.5 | 0 (0.0) | 0 (0.0) | 1 (4.3) | 4 (17.4) | 18 (78.3) | 22 (95.7) |
| Descending aorta | 4.6 ± 0.8 | 0 (0.0) | 0 (0.0) | 3 (15.8) | 1 (5.3) | 15 (78.9) | 16 (84.2) | 4.9 ± 0.3 | 0 (0.0) | 0 (0.0) | 0 (0.0) | 2 (8.7) | 21 (91.3) | 23 (100.0) |
| Abdominal aorta | 4.2 ± 0.8 | 0 (0.0) | 0 (0.0) | 4 (21.1) | 7 (36.8) | 8 (42.1) | 15 (78.9) | 4.7 ± 0.5 | 0 (0.0) | 0 (0.0) | 1 (4.3) | 4 (17.4) | 18 (78.3) | 22 (95.7) |
| Terminal aorta | 4.3 ± 0.7 | 0 (0.0) | 0 (0.0) | 3 (15.8) | 7 (36.8) | 9 (47.4) | 16 (84.2) | 4.6 ± 0.8 | 0 (0.0) | 0 (0.0) | 4 (17.4) | 2 (8.7) | 17 (73.9) | 19 (82.6) |
| Common iliac artery | 4.4 ± 0.8 | 0 (0.0) | 0 (0.0) | 3 (15.8) | 5 (26.3) | 11 (57.9) | 16 (84.2) | 4.5 ± 0.9 | 0 (0.0) | 1 (4.3) | 3 (13.0) | 3 (13.0) | 16 (69.6) | 19 (82.6) |

**Supplemental Table 5. Interobserver agreement for subjective image quality assessment of CCTA and CTAO**

A. CCTA image quality

| Protocol | Patients (n) | Paired evaluation (n) | Complete agreement (%) | Quadratic weighted κ (95% CI)) | Gwet’s AC2 (95% CI) |
| --- | --- | --- | --- | --- | --- |
| Separate scan | 19 | 275 | 92.4 | 0.847 (0.692–0.917) | 0.987 (0.982–0.993) |
| One-scan | 23 | 326 | 92.6 | 0.816 (0.648–0.903) | 0.989 (0.984–0.994) |

B. CTAO quality

| Protocol | Patients (n) | Paired evaluation (n) | Complete agreement (%) | Quadratic weighted κ (95% CI)) | Gwet’s AC2 (95% CI) |
| --- | --- | --- | --- | --- | --- |
| Separate scan | 19 | 114 | 59.6 | 0.667 (0.502–0.784) | 0.971 (0.950–0.987) |
| One-scan | 23 | 138 | 77.5 | 0.552 (0.353–0.759) | 0.975 (0.952–0.991) |

CCTA, coronary computed tomography angiography; CTAO, computed tomography aortography; CI, confidence interval
